# A comprehensive study of the novel 4D hyperchaotic system with self-exited multistability and application in the voice encryption

**DOI:** 10.1038/s41598-024-63779-1

**Published:** 2024-06-06

**Authors:** Khaled Benkouider, Aceng Sambas, Talal Bonny, Wafaa Al Nassan, Issam A. R. Moghrabi, Ibrahim Mohammed Sulaiman, Basim A. Hassan, Mustafa Mamat

**Affiliations:** 1 Department of Electronics, Faculty of Technology, Badji-Mokhtar University, B.P. 12, Sidi Ammar, Annaba, Algeria; 2 Faculty of Informatics and Computing, Universiti Sultan Zainal Abidin (UniSZA), Besut Campus, 22200 Besut, Malaysia; 3 Department of Mechanical Engineering, Universitas Muhammadiyah Tasikmalaya, Tasikmalaya, 46196 Indonesia; 4 Department of Computer Engineering, University of Sharjah, Sharjah, United Arab Emirates; 5 Computer Science Department, School of Arts and Sciences, University of Central Asia, Naryn, Kyrgyz Republic; 6 Department of Information Systems and Technology, Kuwait Technical College, Kuwait city, Kuwait; 7 Institute of Strategic Industrial Decision Modelling (ISIDM), School of Quantitative Sciences, Universiti Utara Malaysia, 06010 Sintok, Kedah Malaysia; 8 Faculty of Education and Arts, Sohar University, Sohar, 311 Oman; 9 College of Computer Science and Mathematics, University of Mosul, Mosul, Iraq; 10 University College Bestari Putera Jaya, Setiu, 22100 Permaisuri, Terengganu Malaysia

**Keywords:** Chaos, Bifurcation, Hyperchaos, Electronic circuit and voice encryption, Engineering, Mathematics and computing, Physics

## Abstract

This paper describes a novel 4-D hyperchaotic system with a high level of complexity. It can produce chaotic, hyperchaotic, periodic, and quasi-periodic behaviors by adjusting its parameters. The study showed that the new system experienced the famous dynamical property of multistability. It can exhibit different coexisting attractors for the same parameter values. Furthermore, by using Lyapunov exponents, bifurcation diagram, equilibrium points’ stability, dissipativity, and phase plots, the study was able to investigate the dynamical features of the proposed system. The mathematical model’s feasibility is proved by applying the corresponding electronic circuit using Multisim software. The study also reveals an interesting and special feature of the system’s offset boosting control. Therefore, the new 4D system is very desirable to use in Chaos-based applications due to its hyperchaotic behavior, multistability, offset boosting property, and easily implementable electronic circuit. Then, the study presents a voice encryption scheme that employs the characteristics of the proposed hyperchaotic system to encrypt a voice signal. The new encryption system is implemented on MATLAB (R2023) to simulate the research findings. Numerous tests are used to measure the efficiency of the developed encryption system against attacks, such as histogram analysis, percent residual deviation (PRD), signal-to-noise ratio (SNR), correlation coefficient (cc), key sensitivity, and NIST randomness test. The simulation findings show how effective our proposed encryption system is and how resilient it is to different cryptographic assaults.

## Introduction

After the seminal work of Lorenz, the construction of models and implementation of circuits for chaotic systems have emerged as an intriguing area of research, driven by the significant potential of chaotic systems in various engineering applications, particularly in secure communications. In terms of Lyapunov exponents, high-dimensional 4D hyperchaos dynamical systems with two positive Lyapunov exponents can produce more unpredictable and complex random signals than 3D chaotic systems, which improves the security of chaos-based communication systems. Consequently, the literature has reported numerous 4-D hyperchaos systems with two positive Lyapunov exponents^1,2^.

In addition, the existence of multistability phenomenon in certain chaotic systems is very interesting. Multistability or the presence of different coexisting attractors for a given set of system parameters allows the system to behave with a high degree of flexibility and this has recently become a fundamental research topic explored by various researchers. For instance, studies by^3,4^ recently investigated some chaotic systems with multistability. Furthermore, a chaotic system that allows for free offset boosting control is attractive for engineering and industrial applications. A prospective chaotic system’s offset boosting control is a crucial component that makes converting a bipolar signal to a unipolar signal or vice versa very helpful. Many studies have been conducted recently on chaotic systems with offset boosting control^5,6^. Therefore, developing a new system with multiple positive Lyapunov exponents, multistability and offset boosting is a challenging, interesting, and very demanding task. Chaotic systems are known for their intricate and unpredictable behavior, making them suitable for generating cryptographic keys and enhancing encryption algorithms^7,8^. Therefore, chaotic systems have been used intensively to improve the security of different information formations, images, voices, and videos. Integrating a chaotic system with encryption systems increases the complexity and makes it more difficult for attackers to decrypt data^9–13^.

Numerous studies delve into hyperchaotic systems, such as the work by Xu et al.^14^, who by featuring torus attractors was able to introduce a novel 4D hyperchaotic system and further demonstrated the presence of the maximum of two positive Lyapunov exponents. In a related vein, Hui et al.^15^ proposed an intricate four-dimensional hyperchaotic system exhibiting a larger key space and more complex dynamic behaviors. Their application extended to image encryption via the hyperchaotic sequence and Secure Hash Algorithm-512 (SHA-512) functions. Fu et al.^16^ developed a 4D hyperchaotic system derived from a 3D Lü chaotic system’s foundation. This system found application in audio encryption employing a cross-XOR algorithm, implemented on the platform of STM32 embedded hardware. Li et al.^17^ introduced a 4D hyperchaotic system incorporating certain exponential term. They explored synchronization issues using an adaptive control law having two inputs. Yang et al.^18^ presented a sequential architecture that converts 3D quadratic system into a basic 6D hyperchaotic system with complex dynamics using linear control. Notably, when the system had a single equilibrium line, they found that at least seven distinct attractors coexisted. Jiang et al.^19^ put forth a brand-new TDCS that exhibits hyperchaotic behavior over the whole parameter range. Their method involved fusing SVD, BP, and the recently created TDCS to create an image cryptosystem with aesthetic significance. Meanwhile, Gu et al.^20^ introduced a novel conservative hyperchaotic system that is non-Hamiltonian in four dimensions. They compared the time sequences produced by the system with previously published works using a complexity analysis of the study. Kong et al.^21^ introduced a novel 5D FOD memristive HNN (FOMHNN) framework aimed at simulating a neuron’s autapse and clarifying electromagnetic induction resulting from electromagnetic radiation. Additionally, they developed a secure image encryption scheme that reduces memory overhead and simplifies hardware implementation significantly. On the other hand, Yu et al.^22^ designed three distinct memristive HNNs, including a 4D memristive synapse weight HNN, and designed an image encryption circuit to allow FPGA to directly encrypt images and transmit them to the IO device.

The main contribution and novelty of this paper is proposing a new 4D dynamical system with two positive Lyapunov exponents, multistability and offset boosting. Dynamical properties of the proposed scheme are studied using LEs, bifurcation diagrams, phase plots, and stability analysis. Different coexisting attractors are displayed. An equivalent electrical circuit is designed using Multisim to validate the proposed system’s feasibility. Finally, offset boosting control is achieved and shown using numerical plots. Moreover, it demonstrates the effectiveness of the properties of the proposed system, which is helpful for chaos-based applications. The paper introduces a novel voice encryption system using the new hyperchaotic system for encrypting voice signals. Then, various tests were applied to determine how effectively the encryption system responded against attacks, including histogram analysis, percent residual deviation (PRD), signal-to-noise ratio (SNR), correlation coefficient (CC), key sensitivity, and NIST randomness test.

## A new 4D hyperchaotic system

**Figure 1:**
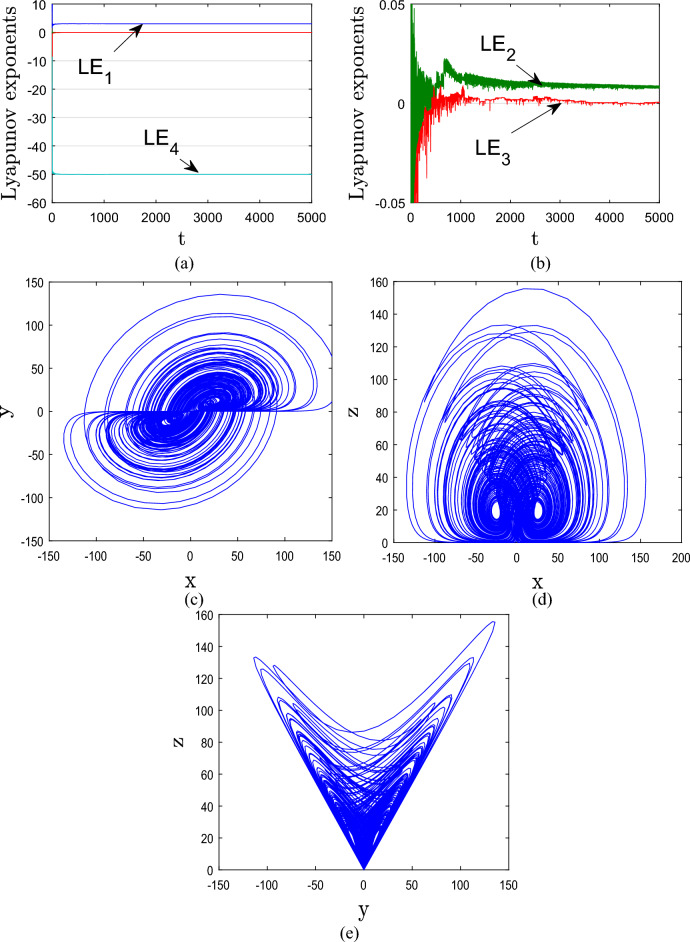
Lyapunov exponents and hyperchaotic attractors of the proposed system (1) for a = 8, b = 40, c = 15, and d = − 0.1

The new 4D hyperchaotic system is defined by the algebraic equations that follows:

1 $$ \left\{ \begin{gathered} \dot{x} = ax - yz + w \hfill \\ \dot{y} = xz - by \hfill \\ \dot{z} = xy - cz \hfill \\ \dot{w} = - y + d \hfill \\ \end{gathered} \right. $$

where the parameters *a, b, c, d* denotes positive constant and *x, y, z, w,* define the state variables. This study aim to establish that system (1) is hyperchaotic by taking the initial state as [10,1,10,1]^T^ and parameters as *a* = 8, *b* = 40, *c* = 15, *d* = − 0.1. In Fig. 1, we generate the hyperchaotic attractors of system (1) using the MATLAB ode45 function.

By choosing system (1)’s previous parameter values, the corresponding Lyapunov exponents are calculated using Wolf’s algorithm^23^ incorporated in MATLAB as:

2 $$ \left\{ \begin{gathered} LE_{1} = 3.036 \hfill \\ LE_{2} = 0.010 \hfill \\ LE_{3} = 0 \hfill \\ LE_{4} = - 50.032 \hfill \\ \end{gathered} \right.\, $$

From (2), System (1) has: *LE*_1,2_ > 0, *LE*_3_ = 0, *LE*_4_ < 0 as depicted in Fig. 1 implying that the system exhibits a hyperchaotic behaviour with two positive Lyapunov exponents, one zero Lyapunov exponents, and one negative Lyapunov exponent. Also, we note that the sum of the Lyapunov exponents is negative, confirming that the proposed 4D system (1) is dissipative.

According to the chaos theory, a high value of Kaplan–Yorke dimension directly corresponds to high complexity of system dynamics. The corresponding Kaplan-Yorke dimension for the suggested system (1) is computed as follows:

3 $$ D_{KY} = 3 + \frac{{LE_{1} + LE_{2} + LE_{3} }}{{\left| {LE_{4} } \right|}} = 3.061 $$

The fractional nature of the Kaplan-Yorke dimension is seen from Eq. (3). Thus, the new 4D system (1) produces intricate hyperchaotic attractors. This section will examine the effects of the parameters and initial conditions on the complexity and features of system (1).

System (1) has two equilibrium points; they are obtained as the following:

4 $$ \begin{gathered} E_{1} = \left[ {\frac{c}{d}\sqrt {\frac{{bd^{2} }}{c}} ,\,d,\,\sqrt {\frac{{bd^{2} }}{c}} ,\,\left( {1 - \frac{ac}{d}} \right)\sqrt {\frac{{bd^{2} }}{c}} } \right] \hfill \\ E_{2} = \left[ { - \frac{c}{d}\sqrt {\frac{{bd^{2} }}{c}} ,\,d,\, - \sqrt {\frac{{bd^{2} }}{c}} ,\,\left( {\frac{ac}{d} - 1} \right)\sqrt {\frac{{bd^{2} }}{c}} } \right] \hfill \\ \end{gathered} $$

By examining the eigenvalues of the Jacobian matrix ${J}_{{E}_{i}}$, the study was able to investigate the stability of the new system (1) at the two equilibrium positions. All equilibrium points are unstable, as Table 1 demonstrates. Therefore, it follows that the 4D system is a member of the self-excited family.

**Table 1:** Eigen value of the new 4d hyperchaotic system.

*E*_*i*_	*J*_*Ei*_ eigenvalues	Stability
*E*_*1*_	λ_1_ = − 54.995, λ_2_ = 7.995, λ_3_ = 0.105, λ_4_ = − 0.105	Unstable
*E*_*2*_	λ_1_ = − 54.995, λ_2_ = 7.995, λ_3,4_ = 0.001 + − 0.105i	Unstable

## Dynamical analysis of the new 4D hyperchaotic system

### Bifurcation analysis

**Figure 2:**
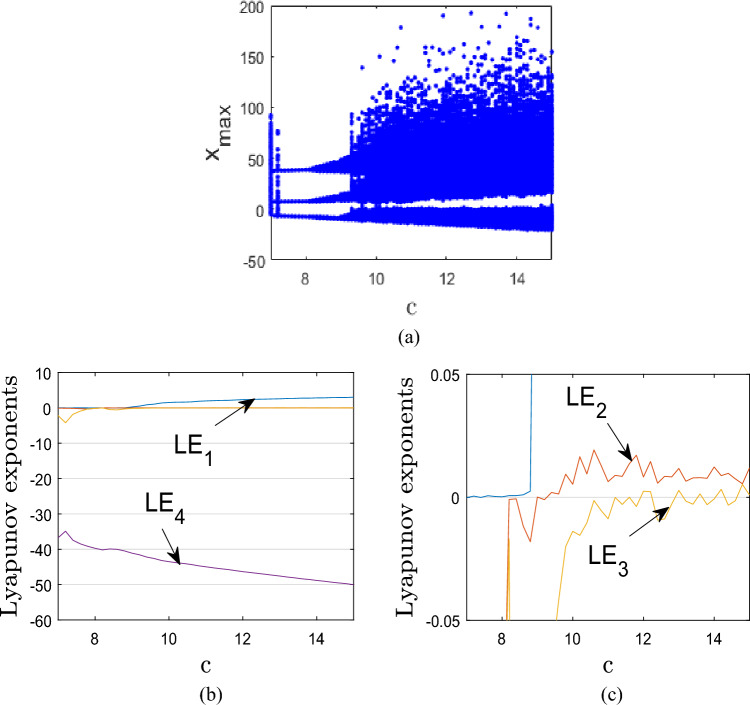
Graphical presentation of bifurcation and LEs spectrum of system (1) for specific interval of the control parameter c.

This section examines the dynamic characteristics of the 4D hyperchaotic system (1) versus the control parameter *c* using phase graphs, bifurcation diagrams, and Lyapunov exponents spectra. Figures 2a and b show the Lyapunov exponents spectrum and the bifurcation diagram of the new system, respectively, when the parameters *a*, *b*, and *d* are fixed at *a* = 8, *b* = 40, and *d* = − 0.1, while the control parameter *c* is adjusted in the range^7,15^.

**Figure 3:**
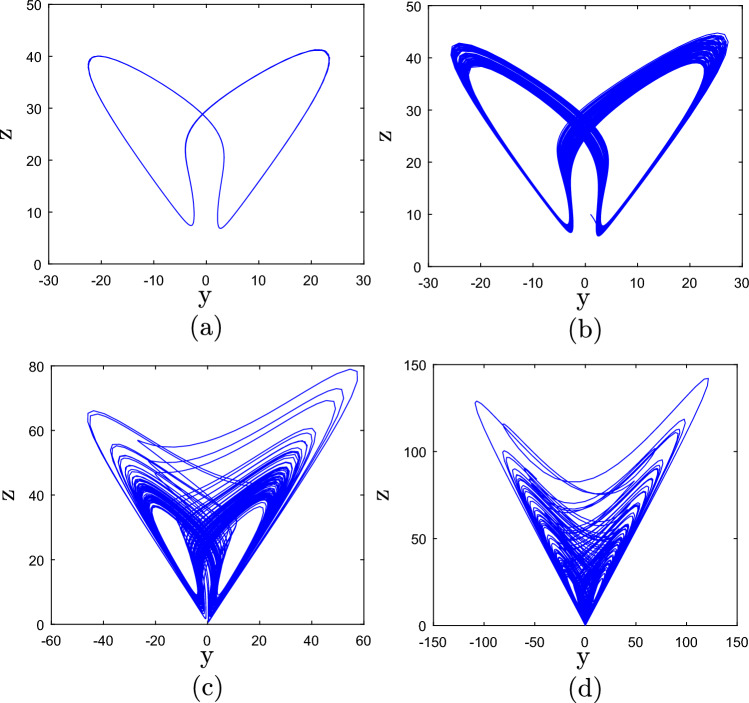
Phase plots of system (1) for specific values of the control parameter c.

When 7 < *c* < 8, system (1) has no positive Lyapunov exponent implying that the system exhibits periodic behaviour as shown in Fig. 3a for *c* = 7. The corresponding *LEs* are: *LE*_1_ = 0, *LE*_2_ = − 0.078, *LE*_3_ =− 2.202, *LE*_4_ = − 36.719.

When 8 < *c* < 9, system (1) has exhibits quasi-periodic behaviour as demonstrated in Fig. 3b for *c* = 8.4. The corresponding *LEs* are: *LE*_1_ = 0, *LE*_2_ = 0, *LE*_3_ = -0.484, *LE*_4_ = − 39.916.

When 9 < *c* < 10, system (1) exhibits chaotic behaviour with one positive LE as depicted in Fig. 3c for *c* = 9.4. The corresponding *LEs* are: *LE*_1_ = 0.847, *LE*_2_ = 0, *LE*_3_ = -0.073, *LE*_4_ = − 42.171.

When 10 < *c* < 15, system (1) has two positive Lyapunov exponents implying that the system exhibits hyperchaotic behavior as presented in Fig. 3d for *c* = 14. The corresponding *LEs* are: *LE*_1_ = 2.818, *LE*_2_ = 0.012, *LE*_3_ = 0, *LE*_4_ = − 48.810.

### Coexisting attractors

**Figure 4:**
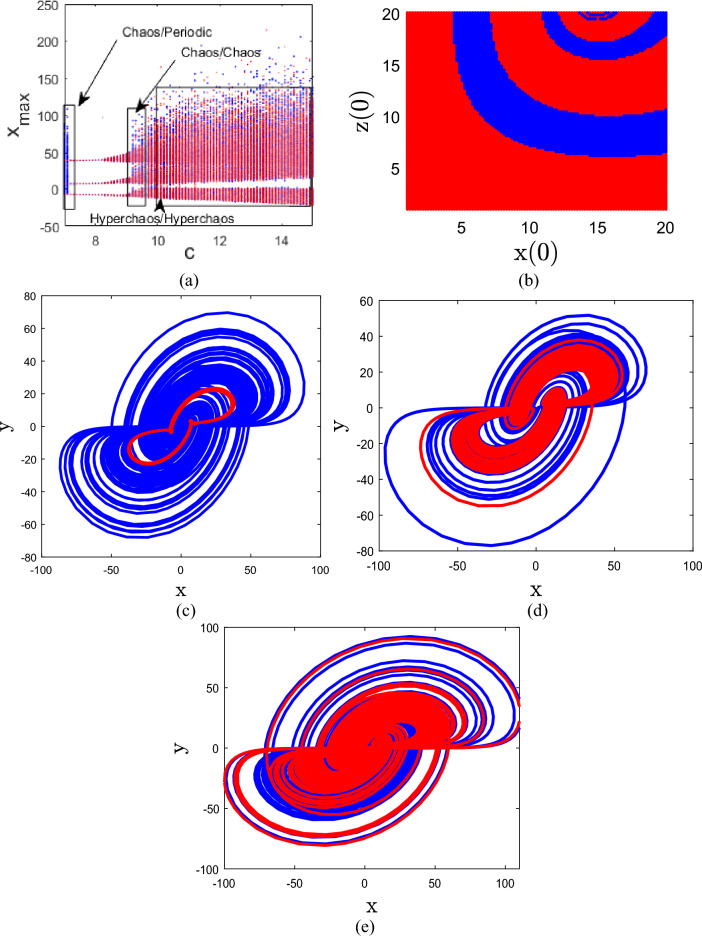
Graphical presentation of system (1): (**a**) coexisting bifurcation diagram for 7 < c < 15, (**b**) basin of attraction in x(0)—z(0) plane with y(0) = w(0) = 1 and c = 7, (**c**) coexistence of one chaotic attractor and one periodic attractor, (**d**) coexisting of two chaotic attractors, (**e**) coexisting of two hyperchaotic attractors.

For varying parameter values, the 4D system (1) exhibits the coexistence of one periodic attractor and one chaotic attractor, the coexistence of two hyperchaotic attractors, or the coexistence of two chaotic attractors, as demonstrated by the coexisting bifurcation diagram, basin of attraction, and phase plots. Assume that *Y*_01_ = (1, 1, 1, 1) and *Y*_02_ = (10, 1, 10, 1) are two distinct starting points for the new 4D system (1). We will use blue for the state orbit associated with *Y*_*01*_ and red for the state orbit associated with *Y*_*02*_. Figure 4a illustrates the bifurcation diagram of system (1) versus parameters c, calculated and plotted starting from *Y*_01_ and *Y*_02_, showing that the new system can generate coexisting attractors. Additionally, Fig. 4b illustrates the basin of attraction for system (1) plotted in the *x*(0)-*z*(0) plane, highlighting regions associated with distinct behaviors. The blue region represents the basin of attraction for periodic behavior, where trajectories converge to periodic orbits, while the red regions denote areas where chaotic dynamics emerge.

Adjusting the values of *a* = 8, *b* = 40, *c* = 7, and *d* = − 0.1, as seen in Fig. 4c, the 4D system (1) exhibits the coexistence of one chaotic attractor and one periodic attractor. Setting *a* = 8, *b* = 40, *c* = 9.4, and *d* = − 0.1 as the parameters, as shown in Fig. 4d, the 4D system (1) exhibits two coexisting chaotic attractors. Further adjusting the values to *a* = 8, *b* = 40, *c* = 10, and *d* = − 0.1, as depicted in Fig. 4e, the 4D system (1) exhibits two coexisting hyperchaotic attractors.

### Offset boosting control

**Figure 5:**
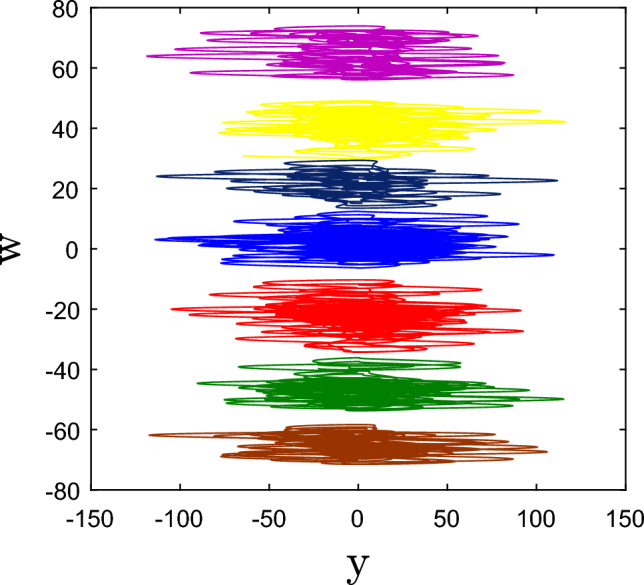
The plot of y-w hyperchaotic attractor of system (1) boosted with various values of offset boosting parameter k = 0 (blue), 10 (red), − 10 (black) 20 (green), − 20 (yellow), 30 (brown), and − 30 (magenta).

Since the fourth variable of system (1), $w$ appears just in the first equation, the proposed 4-D scheme is a variable-boosting hyperchaotic system with controlled $w.$ As a result, $w$ can be boosted by replacing $w$ with $(w + k)$. System (1)’s first differential equation can be rewritten as follows:

5 $$ \dot{x} = ax - yz + (w + k) $$

where $k$ is a controller for offset boosting. Figure 5 depicts several positions of hyperchaotic attractors boosted on different $k$ in the *x–y* plane. For a positive value of $k,$ the attractors are boosted in the negative direction while for a negative value of $k,$ the attractors are boosted in the positive direction. This special property has a numerous application in chaos-based cryptography and other engineering domains^24–27^

## Circuit design

**Figure 6:**
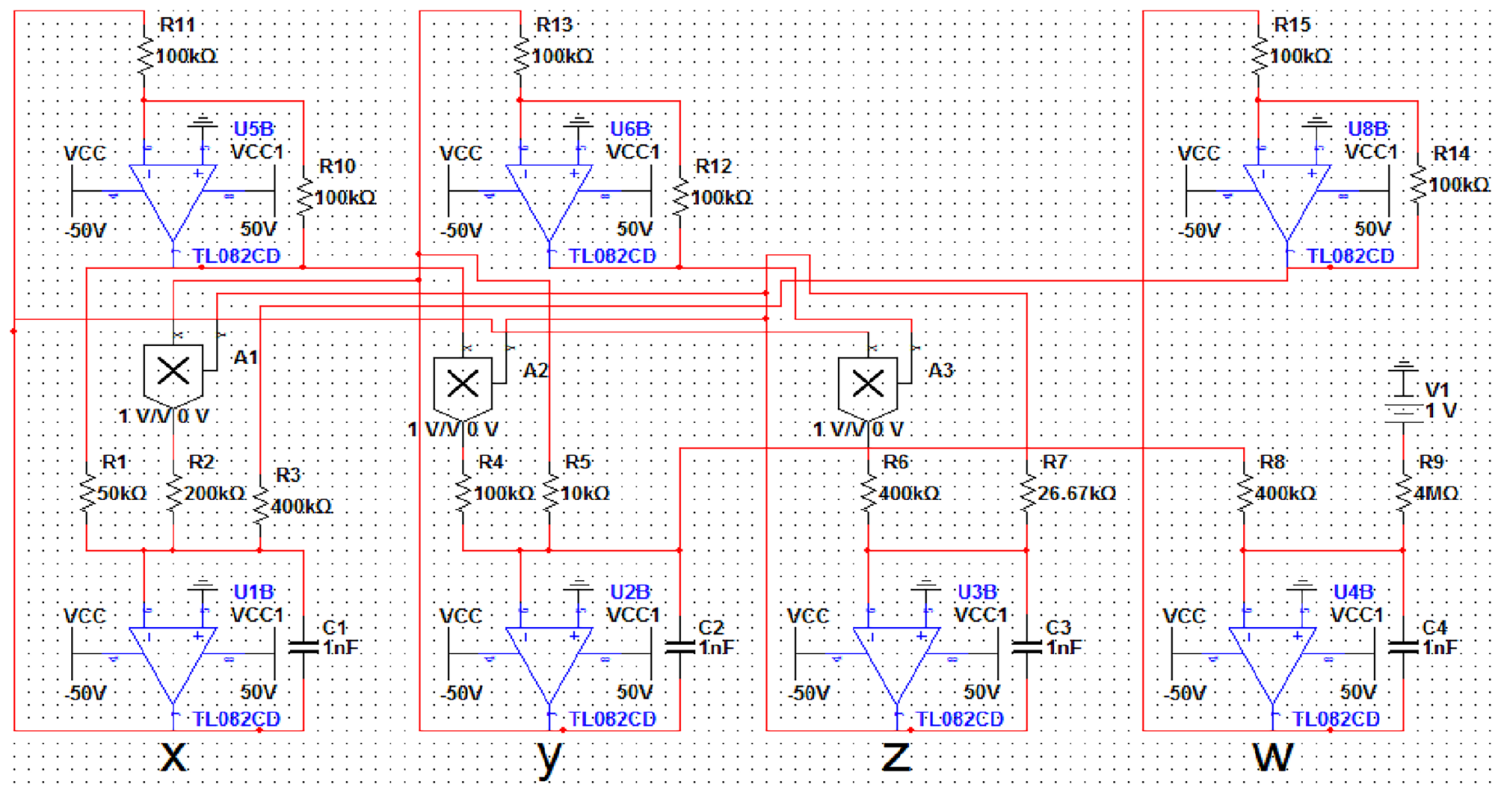
The schematic of the circuit.

Multisim software is used to implement the analog circuit of the proposed 4D hyperchaotic system (1). Figure 6 displays the circuit schematic of the 4D hyperchaotic system. The circuit used in this study includes linear resistors, capacitors, an operational amplifier (TL082CD), and a multiplier (AD633). A power supply of ± 15 V is used. Circuit functions including addition, subtraction, and integration are performed by the operational amplifier. In the meantime, the multiplier unifies the four state variables into a coherent whole while capturing the nonlinearity of the system.

The following corresponding circuital equations of proposed scheme (1) is derived by applying Kirchhoff’s laws to the circuit in Fig. 6, where:

6 $$ \left\{ \begin{gathered} \dot{x} = \frac{1}{{R_{1} C_{1} }}x - \frac{1}{{R_{2} C_{1} }}yz + \frac{1}{{R_{3} C_{1} }}w \hfill \\ \dot{y} = \frac{1}{{R_{4} C_{2} }}xz - \frac{1}{{R_{5} C_{2} }}y \hfill \\ \dot{z} = \frac{1}{{R_{6} C_{3} }}xy - \frac{1}{{R_{7} C_{3} }}z \hfill \\ \dot{w} = - \frac{1}{{R_{8} C_{4} }}y - \frac{1}{{R_{7} C_{4} }}V_{1} \hfill \\ \end{gathered} \right. $$

7 $$ \left\{ \begin{gathered} C_{1} = C_{2} = C_{3} = C_{4} = 1nF \hfill \\ R_{1} = 50k\Omega \hfill \\ R_{2} = R_{3} = R_{4} = R_{6} = R_{8} = 400k\Omega \hfill \\ R_{5} = 10k\Omega ,\,\,\,R_{7} = 26.67k\Omega \hfill \\ R_{9} = 4M\Omega \hfill \\ R_{i} = 100k\Omega ,\,\,i = 10,\,......,\,15 \hfill \\ \end{gathered} \right. $$

**Figure 7:**
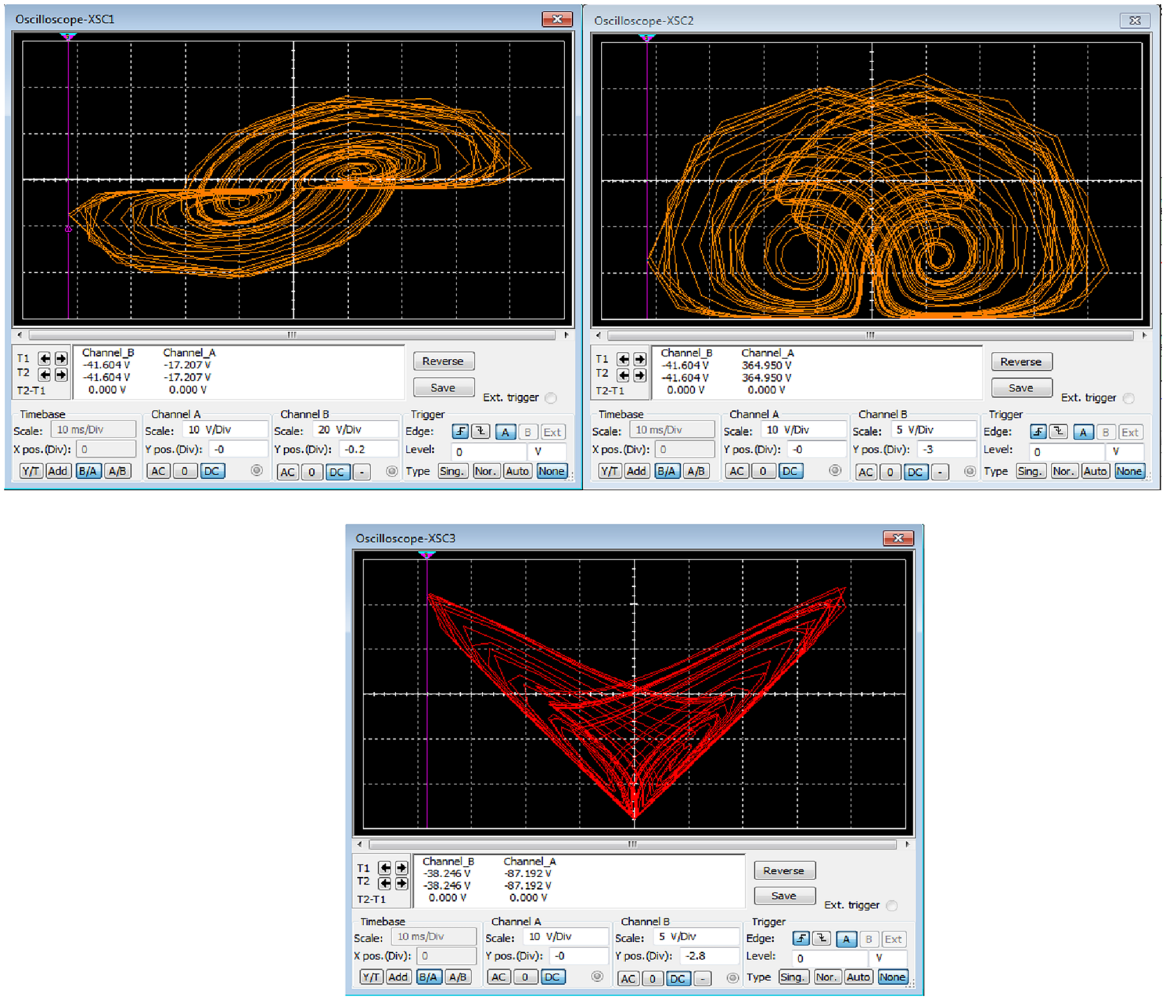
Experimental phase plots of the new hyperchaotic system.

The agreement between the outcomes derived from numerical simulation software Matlab (See Fig. 1) and the electronic circuit simulation software Multisim (See Fig. 7) establishes strong validation for both hyperchaotic attractors of the novel hyperchaotic systems.

## Voice encryption algorithm

**Figure 8:**
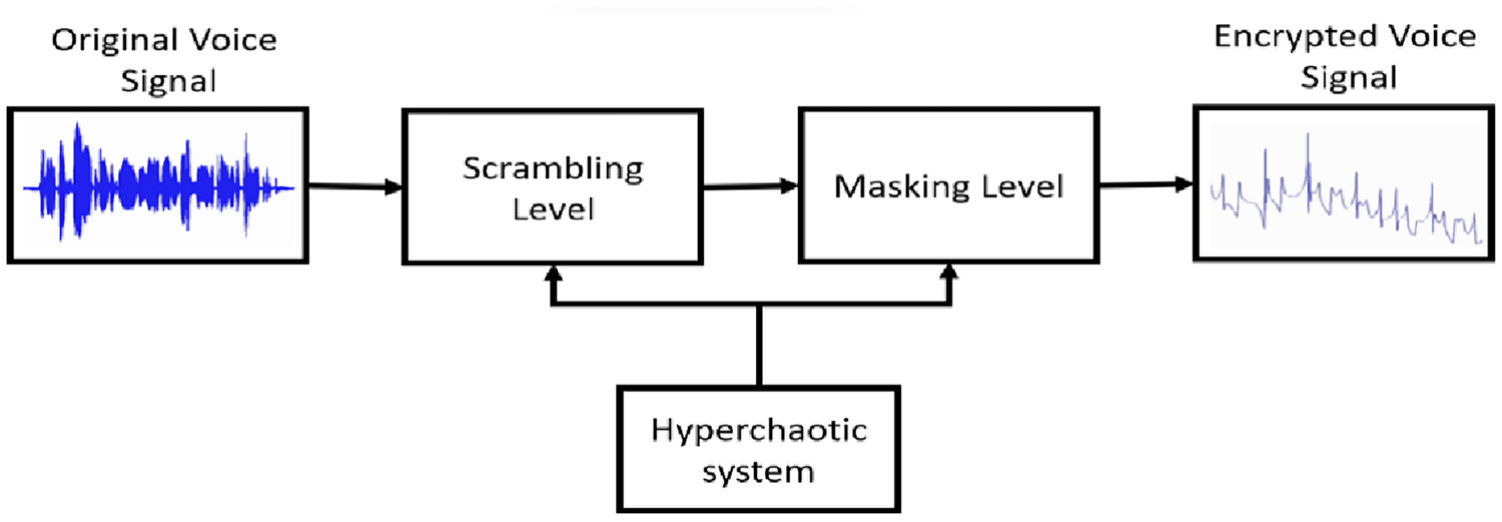
The voice encryption scheme using the new hyperchaotic system.

**Figure 9:**
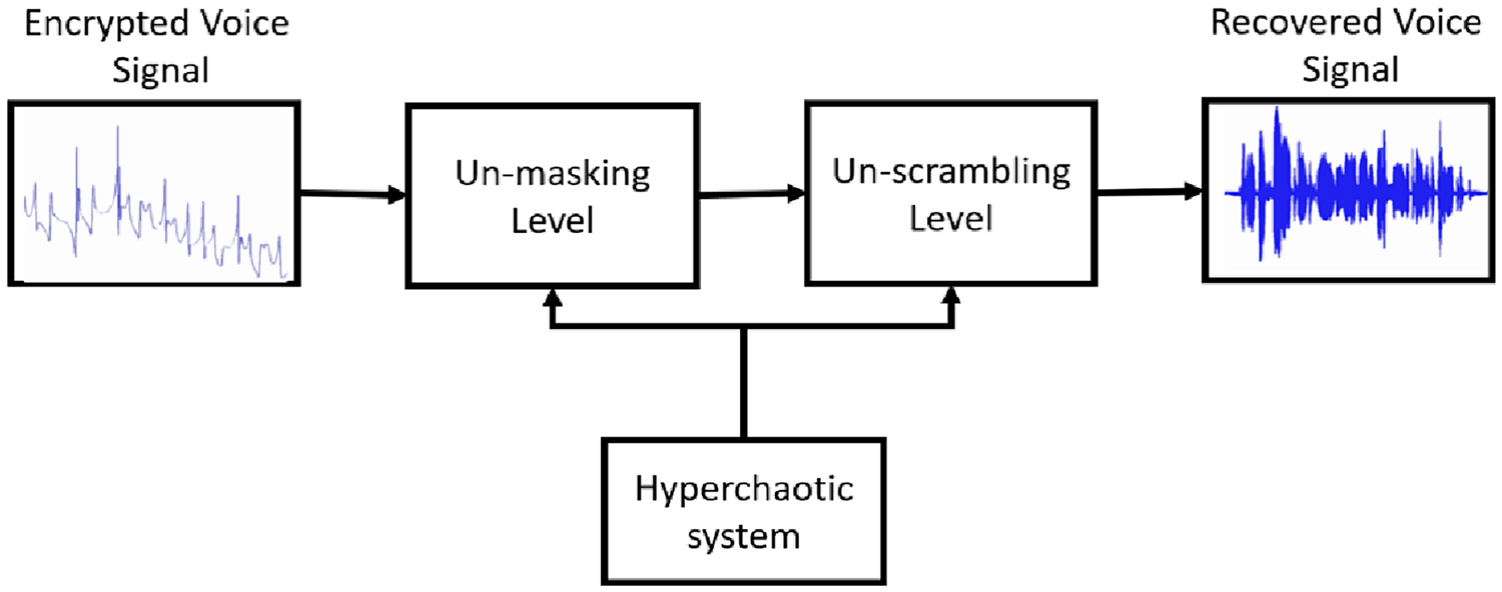
The voice decryption scheme using the new hyperchaotic system.

The block diagrams of voice encryption\decryption using the new hyperchaotic process are demonstrated in Fig. 8 and Fig. 9 respectively.

As displayed in Fig. 8, the encryption process contains two levels: scrambling level and masking level, where both levels depend on the sequence generated by the proposed scheme. Chaotic scrambling is the process of transforming the original voice signal based on a particular algorithm^28–30^. This transformation can be achieved through various techniques, including mathematical transformations, encryption algorithms, or chaotic systems. In our system, the voice signal is scrambled using the *x, y, z,* and *w* sequences generated by the new hyperchaotic system according to the following equation:

8 $${V}_{s}=v\left(t\right)+scramblingStrength*({s}_{1}* x\left(t\right){.}^{p}+ {s}_{2} * y{\left(t\right).}^{p2}+{s}_{3}* z{\left(t\right).}^{p3} + {s}_{4} * w\left(t\right){.}^{p4})$$

The scrambling equation introduces a more sophisticated algorithm by integrating chaos and nonlinearity into the scrambling process. Where *V*_s_ is the scrambled voice signal, *v*(*t*) is the original voice signal *s*_1_, *s*_2_, *s*_3_, and *s*_4_ represent coefficients, and *p*_1_, *p*_2_, *p*_3_, and *p*_4_, represent the power terms associated with each state variable *w*(*t*), *z*(*t*), *y*(*t*), and *x*(*t*). The power terms add an element of nonlinearity to the scrambling process, making it more complex and potentially enhancing the algorithm’s security. Adjusting the power terms allows for fine-tuning the scrambling strength and the degree of nonlinearity introduced into the signal. Where the scrambling strength is set to 0.9, and the values of *p*^1^  = *p*^2^ = *p*^3^  = *p*^4^ = 1. The secret keys used in the encryption process are *s*^1^  = *s*^2^ ​= *s*^3^ = *s*^4^  = 1.

Then, to add a complexity to the encrypted signal, the resulted scrambled signal *V*_s_(*t*) is further masked using the state variables generated from the new scheme producing the encrypted voice signal *V*_e_(*t*) as follows:

9 $${V}_{e}\left(t\right)={V}_{s}\left(t\right)+(x\left(t\right)+y\left(t\right)+z\left(t\right)+w\left(t\right))$$

In masking level, the complete state variables are used for masking the signal to increase the security level of the proposed system.

### Decryption process

The decryption procedure is the vice versa of the encryption procedure, it involves generating an unmask signal by subtracting the same state variables from the encrypted signal producing the signal *V*_d_(*t*) as follows:

10 $${V}_{d}\left(t\right)={V}_{e}\left(t\right)-(x\left(t\right)+y\left(t\right)+z\left(t\right)+w\left(t\right)$$

The produced signal is then unscrambled according to the following equation:

11 $${V}_{r}={V}_{d}\left(t\right)-scramblingStrength*({s}_{1}*x\left(t\right){.}^{p}+{s}_{2}*y{\left(t\right).}^{p2}+{s}_{3}*z{\left(t\right).}^{p3}+{s}_{4} *w\left(t\right){.}^{p4})$$

The resulting signal *V*_r_(*t*), represents the recovered voice signal. Assuming that the hyperchaotic systems in both the encryption and decryption systems are identical, have the same initial condition, and synchronized. The recovered signal *V*_r_(*t*) is like the original voice signal *v*(*t*). In our system the values of coefficients terms are 0.1 for *s*_1_, *s*_2_, and *s*_3_, and 1 for *s*_4._ While the values of the power terms are 1 for *p*_1_, *p*_2_, *p*_3_, and *p*_4_, and 0.5 for the scrambling strength term.

### Numerical simulation and security analysis

MATLAB program was used to acquire the simulation results. The suggested system’s effectiveness and security were evaluated using various tests, including waveform analysis, PRD, SNR, and correlation measurements. The voice signals use eight quantization bits at 8000 Hz.

**Figure 10:**
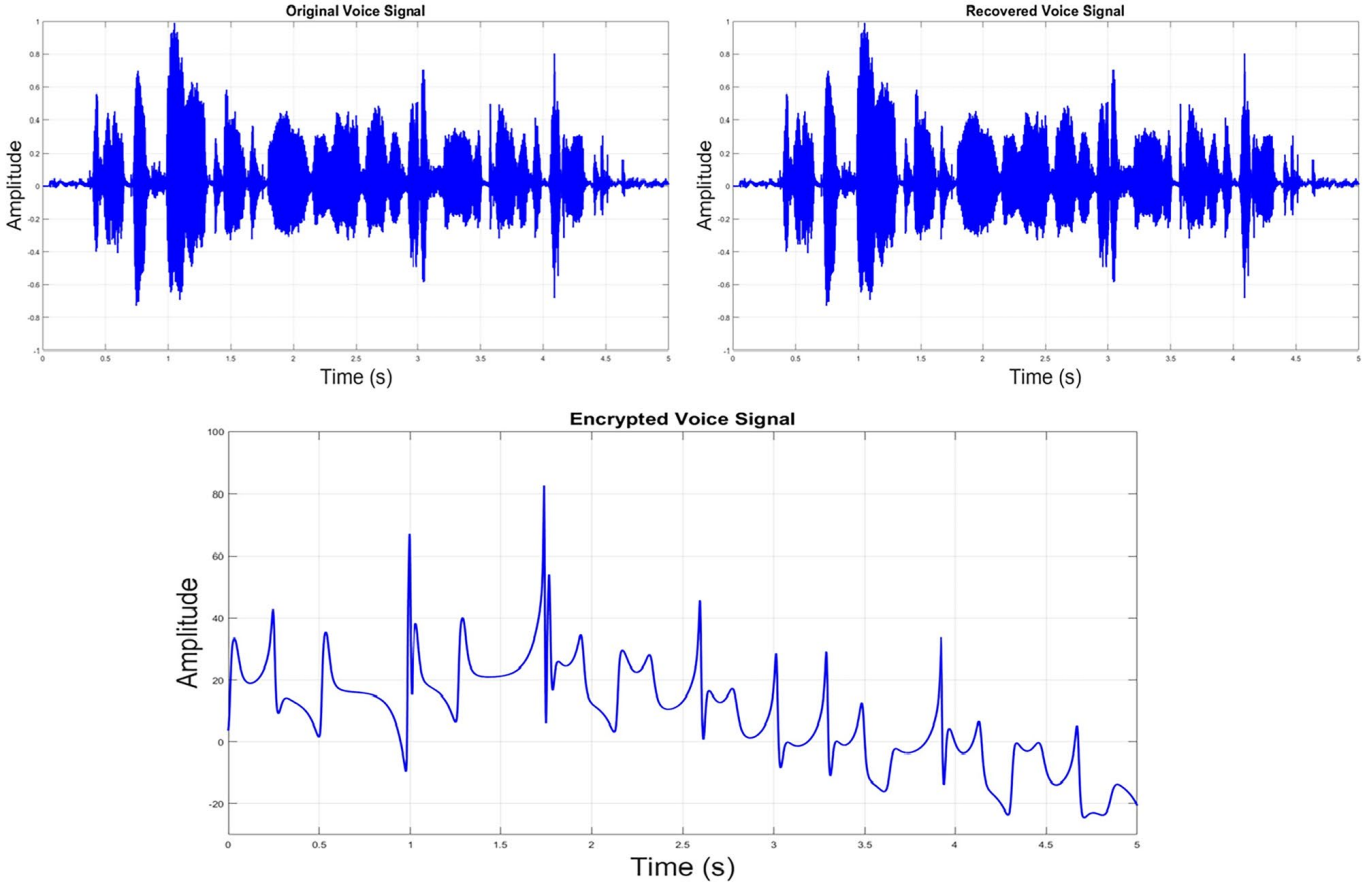
Waveform plots for the encrypted, recovered, and original voice signals.

The waveforms obtained from the proposed encryption system are illustrated in Fig. 10, the recovered, encrypted, and original voice signals, where the original voice transmission is entirely altered by the encrypted signal. Meanwhile, the recovered and original signals are identical.

**Figure 11:**
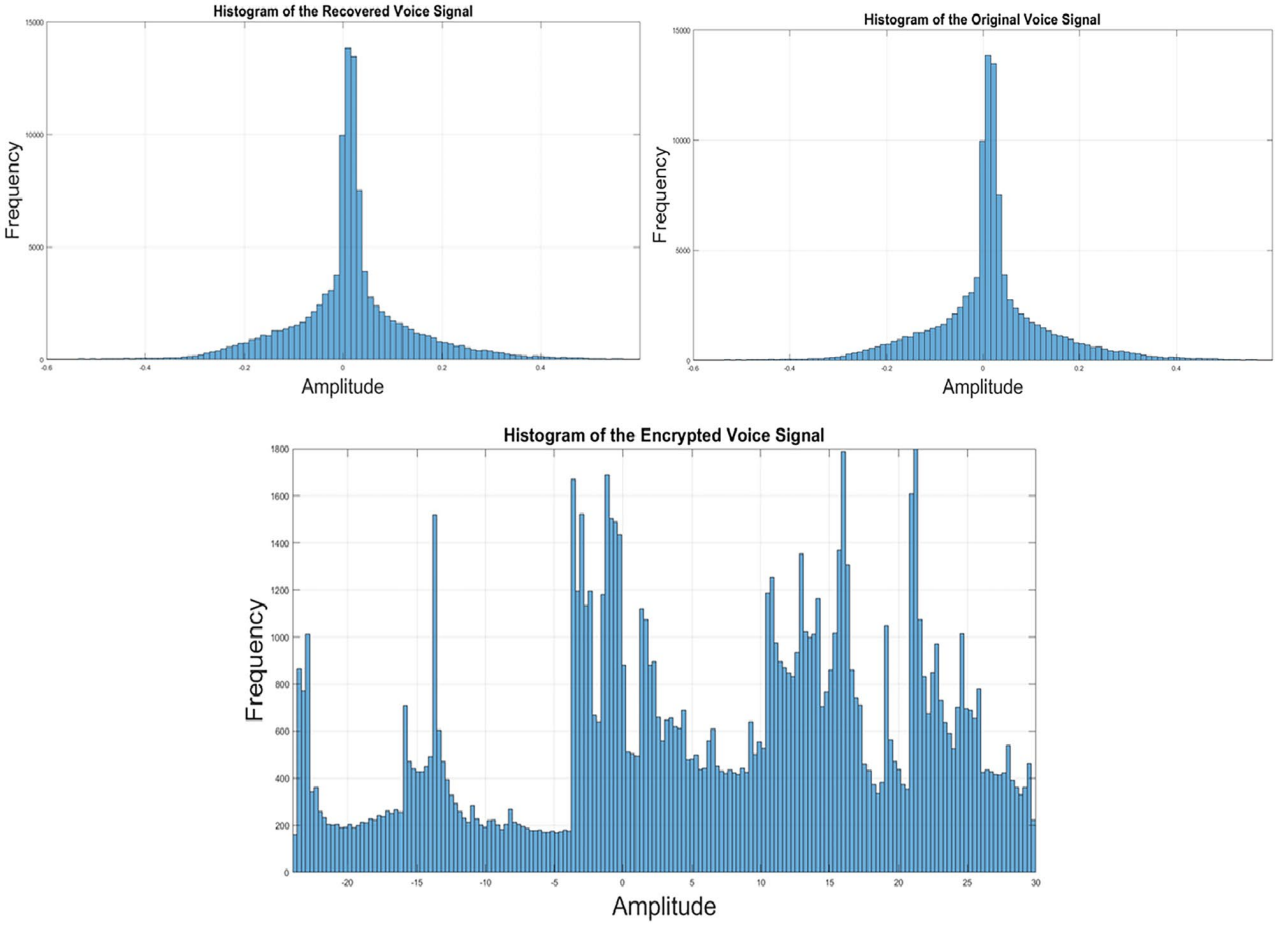
The histograms for the encrypted, recovered, and original voice signals.

Figure 11 depicts the histograms of the encrypted, original, and recovered voice signals. The distributed histogram indicates the randomness of the encrypted voice signal, a stark contrast to the histogram of the original and recovered voice signals, which exhibits a normal distribution, rendering it susceptible to attacks. The analysis reveals that our proposed algorithm provides robust security against various statistical attacks, affirming its efficacy in safeguarding voice communication.

#### Statistical tests

The Percentage Residual Deviation (PRD), Signal Noise Ratio (SNR), and Correlation Coefficient (CC) are employed to analyse the proposed scheme’s immunity against statistical intruders^31–33^. Table 2 displays the values that were computed for various voice signals. The (PRD) is a statistical tool used to measure the deviation between encrypted and original audio signals. Low PRD values suggest a similarity between encrypted and original signals, indicating high fidelity and minimal distortion. Conversely, high PRD values imply significant differences, potentially indicating a decline in signal integrity and increased distortion. Table 2 provides the computed percent residual deviation values for a range of original and encrypted voice signals.

**Table 2:** The measurements of statistical metrics for different voice samples.

Voice	PRD	SNR	Correlation (cc)
Voice-1	1.2366e + 05	-61.8448	0.0005
Voice-2	1.5318e + 05	-63.7043	0.0011
Voice-3	1.6546e + 05	-64.3737	0.0013
Voice-4	1.4497e + 05	-63.2255	0.001
Voice-5	1.4331e + 05	-63.1255	0.0014
Voice-6	1.4320e + 05	-63.1189	0.0016

One of the widely considered objective metrics for assessing the strength of the original audio signal is the signal–noise ratio (SNR). The measurements of the SNR in Table 2 are highly negative, indicating an enormous quality of the encrypted speech signals. The correlation coefficient is a numerical correlation measure between -1 and 1. Table 2 provides the calculation for various speech signals. The small value of the (CC) obtained demonstrates how severely jumbled the encrypted signal is in comparison to the original voice signal. The higher PRD values suggest a significant deviation between the encrypted and original signals. The large negative SNR value indicates that the noise power is higher than the signal power, which makes it difficult to detect. On the other hand, near-zero correlation values imply a reduced similarity between the encrypted and original signals.

#### Key sensitivity

**Figure 12:**
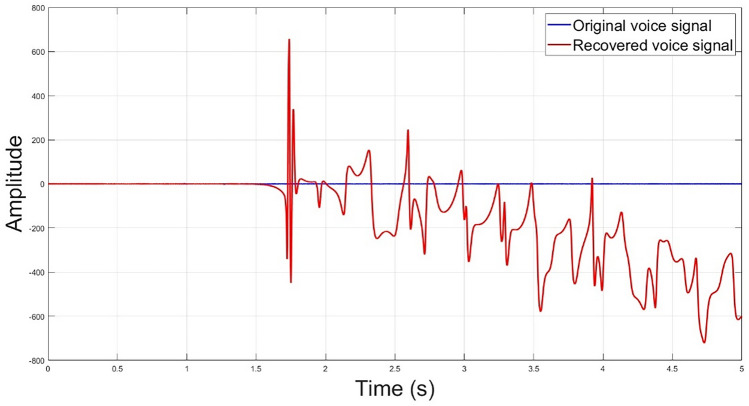
Decrypted speech signal with a bit of change in the initial values × 0.

A key sensitivity analysis has been conducted to assess the responsiveness of the new encryption scheme to slight variations in the key values. A small change in one key, for example, the initial value of the *x* state variable is changed by 0.000000000000001, will entirely deviate the decrypted signal, as shown in Fig. 12, which reflects the immunity of the proposed encryption system against attacks.

#### NIST statistical randomness tests

Utilizing the NIST 800–22 test package, which is provided by the US National Institute of Standards and Technology, we examined the randomness of the encrypted speech signal in this test. This research primarily aims to test the randomness of encrypted and original voice signals. As indicated in Table 3, the tests were used to investigate the degree of randomness of each signal. The bit-stream of the original speech signal passed only 2 of the NIST tests. The results also suggest that the encrypted voice signal performs favorably in several statistical tests, meeting the criteria for randomness and passing certain NIST assessments.

**Table 3:** The outcome of NIST statistical test for encrypted and original voice signals.

Name	Original voice signal	Encrypted voice signal
random excursions	F	P
random excursions variant	F	P
Linear complexity	P	P
Serial	F	F
Universal	F	P
Overlapping template	P	P
Non-overlapping template	P	P
Fast Fourier transform	F	P
Rank	F	F
Longest Run	F	P
Runs	F	P
Approximate entropy	F	P
Cumulative sum	F	P
Block Frequency	F	P
Frequency	F	P

*F* denotes fail, *P* denotes pass.

## Conclusion

This article presents a novel 4-D hyperchaotic system characterized by a high level of complexity, capable of exhibiting periodic, quasi-periodic, chaotic, and hyperchaotic behaviors through parameter adjustments. The system demonstrates the well-known dynamical property of multistability, showcasing various coexisting attractors for identical parameter values. The dynamical characteristics of the proposed system are explored via bifurcation diagrams, Lyapunov exponents, stability analysis of equilibrium points, dissipativity, and phase plots. To validate the mathematical model, Multisim software is used to implement an electronic circuit. The system’s offset boosting control capability is highlighted as an attractive and unique feature. Consequently, the new found 4D system proves to be highly advantageous for applications relying on chaos, given its hyperchaotic behavior, multistability, offset boosting property, and the ease with which its electronic circuit can be implemented. We conducted comprehensive tests, including histogram analysis, PRD, SNR, CC, key sensitivity, and NIST randomness, to evaluate its effectiveness against attacks. Our findings demonstrate robust performance, highlighting the system’s capability to safeguard voice signals effectively. This work advances cryptography through innovative encryption methods tailored for voice communication security. In future research, we will make enhancements to the voice encryption system to bolster its resilience against various attacks. This could involve investigating advanced encryption algorithms, incorporating additional layers of security, or exploring techniques from other domains.

## Data Availability

The data that support the findings of this study are available from the corresponding author on reasonable request.
